# Myocardial Infarct Size Reduction Provided by Local and Remote Ischaemic Preconditioning: References Values from the Hatter Cardiovascular Institute

**DOI:** 10.1007/s10557-018-6788-8

**Published:** 2018-04-14

**Authors:** Xavier Rossello, Zhenhe He, Derek M. Yellon

**Affiliations:** 1 Centro Nacional de Investigaciones Cardiovasculares Carlos III (CNIC), Madrid, Spain; 2 CIBER de enfermedades CardioVasculares, Madrid, Spain; 3 The Hatter Cardiovascular Institute, University College London, 67 Chenies Mews, London, WC1E 6HX UK

**Keywords:** Cardioprotection, Ischaemic preconditioning, Remote ischaemic preconditioning, Myocardial infarction, Ischaemia/reperfusion injury

## Abstract

**Purpose:**

To accurately estimate the effect size of both local or classic ischaemic preconditioning (IPC) and remote ischaemic preconditioning (RIPC) using a pooling data set of 91 animals.

**Methods:**

We combined all the available mouse data collected from our Institute over the last 3 years regarding (i) local IPC (4 cycles of 5 min of global ischaemia/reperfusion injury, IRI, followed by 35-min ischaemia and 2-h reperfusion) in the Langendorff-isolated perfused mouse heart model and (ii) RIPC (3 cycles of 5 min of limb occlusion followed by 40-min ischaemia and 2-h reperfusion) in the in vivo mouse model.

**Results:**

Five independent experiments containing 27 control and 29 IPC mice were used to estimate the overall (i) local IPC effect, which reduced infarct size in the ex-vivo setting by a mean difference of 24.1% (95% CI 19.5, 28.6%) when compared to untreated controls (*P* < 0.001) and for (ii) RIPC, three independent experiments including data for 16 control and 19 RIPC mice were used to estimate that RIPC diminished infarct size in the in-vivo setting by a mean difference of 20.8% (95% CI 14.7, 26.9%) when compared to controls (*P* < 0.001).

**Conclusions:**

Using a significant animal dataset, we found that local IPC reduces myocardial infarct size by 24.1% and RIPC by 20.8% in the ex vivo and in vivo mouse models of IRI, respectively. These differences may be used as reference values to either establish positive controls or to determine by how much myocardial infarct size can be reduced by novel cardioprotective interventions following an IRI insult.

## Introduction

In many countries, basic animal research receives more funding than clinical research on the assumption that it would benefit humans in the long-run [1]. Animal experiments are pivotal to decide what treatment should be taken forward in clinical trials, but the reported treatment effect need to be valid and precise. Biased or imprecise results from animal experiments may result in exposing patients to unnecessary risks and wasting scarce research funds. There is a need to report accurate estimates of treatment effects, not only as the previous step to translate treatments to the clinical setting, but also to be used as a landmark for future animal studies. Repeatable, reliable, and valid treatment effect estimates are the starting point for many researchers who want to investigate any intervention.

Targeting myocardial injury that paradoxically occurs with the acute reperfusion of ischaemic myocardium remains one of the top ten unmet clinical needs in cardiology [2]. Although myocardial reperfusion is needed to salvage viable myocardium in patients presenting with acute myocardial infarction (AMI), it comes at a price in what is known as reperfusion-induced injury (IRI). Therapies aimed to protect the heart against IRI, known as cardioprotective therapies [3], include local ischaemic preconditioning (IPC), whereby brief cycles of coronary occlusion and reperfusion elicit protection from a prolonged IRI. This procedure has emerged as the paradigm of cardioprotection [3] in the animal setting being highly reproducible between laboratories and across species [4]. Both local IPC and remote ischaemic preconditioning (RIPC) have become pivotal in the assessment of novel cardioprotective interventions. First, they are used as positive controls when testing for other cardioprotective therapies. Second, they have been used to unveil the molecular architecture behind the protection against IRI, as it is believed that most cardioprotective interventions share common signalling pathways.

Whilst it is well known that both IPC and RIPC protect the heart against IRI, many unanswered questions remain open to debate. For instance, no consensus has been reached on how many cycles should be applied for optimal protection and what their duration and timing should be [5], or to which extent the interval between preconditioning stimulus and the index ischaemia affects the outcome. There is also little evidence on whether local IPC or RIPC provide similar treatment size, and most of this evidence comes from meta-analyses using aggregated data in exceedingly heterogeneous experimental conditions [4, 6]. In this small study, we combined all the available animal data collected over the last 3 years regarding the use of IPC in the Langendorff perfused isolated mouse heart model and RIPC in the in vivo mouse model from our Institution. The aim is to use this pool of more than 90 animals to provide accurate estimate of the effect size of both IPC and RIPC.

## Methods

All work was conducted in accordance with the Guidelines on the Operation of the Animals (Scientific Procedures) Act 1986, published by The Stationery Office (London, UK), conforming with National Institute of Health Guidelines for the Care and Use of Laboratory Animals.

### Data Acquisition

**Fig. 1:**
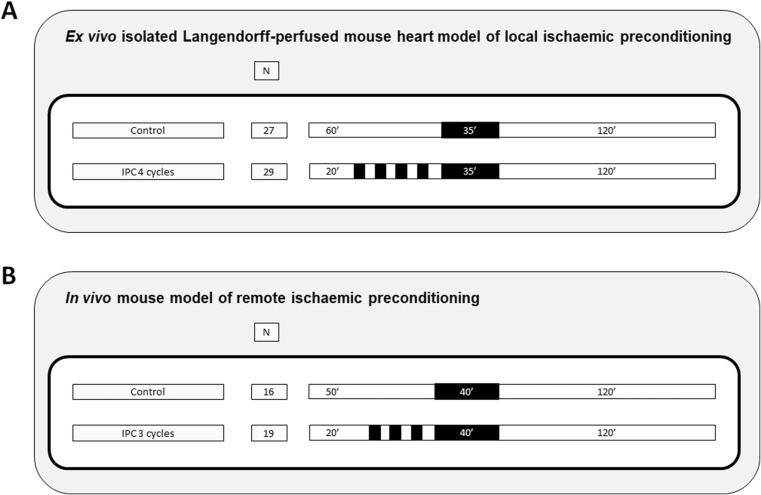
Study protocols to assess the impact on infarct size of ischaemic preconditioning either applied locally in the ex vivo model (**a**) or remotely in vivo model (**b**)

We constructed a common data set by merging the individual experimental data captured over a period of 3 years by two independent researchers, one focused on IPC in the ex vivo mouse model and the other one focused on RIPC in the in vivo mouse model. Using data from five separate sets of IPC ex vivo experiments, 56 animals were randomized to either IPC (4 cycles of 5 min of global IRI followed by 35-min ischaemia and 2-h reperfusion, *n* = 29 animals) or control (40 min of buffer perfusion followed by 35-min ischaemia and 2-h reperfusion, *n* = 27 animals). Using data from three separate sets of RIPC in vivo experiments, 35 animals were randomized to either RIPC (3 cycles of 5 min of limb occlusion followed by 40-min ischaemia and 2-h reperfusion, *n* = 19 animals) or control (sham procedure followed by 40-min ischaemia and 2-h reperfusion, *n* = 16 animals) (Fig. 1). To avoid publication bias, we used all animal data available, regardless of whether these were used in previous peer review publications or as part of ongoing studies [7–9].

### Ex Vivo Isolated Langendorff-Perfused Mouse Heart Model of Acute Myocardial Infarction

Heart isolation and Langendorff perfusion were carried out with filtered modified Krebs-Henseleit buffer (composed of 118 mmol/L NaCl, 25 mmol/L NaHCO_3_, 11 mmol/L glucose, 4.7 mmol/L KCl, 1.22 mmol/L MgSO_4_.7H_2_0, 1.21 mmol/L KH_2_PO_4_, and 1.84 mmol/L CaCl_2_.2H_2_0) aerated with a mixture of O2 (95%) and CO2 (5%) to maintain pH at 7.40 ± 0.3, as previously described [10]. Previously, mice were given terminal anesthesia through an intraperitoneal injection of 60 mg/kg sodium pentobarbitone and anticoagulation (100 IU heparin). Hearts were then harvested and immediately cannulated through a 21-gauge cannula whilst submerged in ice-cold modified Krebs-Henseleit buffer to be eventually perfused on a murine Langendorff perfusion apparatus at 80 mmHg pressure. There were three predefined exclusion criteria: (1) 4 min exceed between heart removal and the Langendorff-perfusion starting time, (2) temperature outside the range of 37 ± 0.5 °C, and (3) isolated heart flow rate of either less than 1 mL/min or more than 6.5 mL/min on the Langendorff preparation during the 20-min stabilization period. After evaluating for exclusion criteria this stabilization period, hearts were either allocated to IPC treatment or control and subsequently subjected to 35 min of ischaemia and 120 min of reperfusion.

### In Vivo Murine Model of Acute Myocardial Infarction

C57Bl/6 mice were anaesthetized by intraperitoneal injection of 80 mg/kg pentobarbitone at a concentration of 20 mg/ml in 0.9% (*w*/*v*) saline and maintained at 36.5 ± 0.5 °C on a heating mat. Surgery was started after confirming the abolishment of pedal and tail reflexes and depth of anaesthesia was monitored throughout. Mice were intubated using a 19G cannula and ventilated with room air using a MiniVent, type 845, Small Animal Ventilator (Harvard Apparatus, Kent, UK), at a flow rate of 1.0 l/min with 2 cmH2O PEEP, stroke volume 200 μl at 130 strokes/min. The left anterior descending (LAD) coronary artery was occluded in all mice (verified by ST elevation, hypokinesia and pallor) for 40 min followed by 2 h reperfusion.

RIPC was induced using a 6-mm lumen custom vascular occluder (Kent Scientific, Torrington, CT, USA) around the right hindlimb inflated to 250 mmHg to induce 3 cycles of 5-min ischaemia, followed by 5-min reperfusion after each cycle. Blood flow was visualized in the hindlimb during RIPC using a FLPI-2 laser speckle contrast blood flow imager (Moor Instruments, Axminster, United Kingdom), which delivers images at high time and spatial resolution. Hair was first removed from both hindlimbs using Veet depilation. Full-frame images were recorded at 5 Hz and then subsequently analysed by averaging the image intensity over an area of interest encompassing the entire exposed upper limb. Limb muscle pO2 was measured regularly using a bare-fibre phosphorescent sensor connected to an Oxylite™ monitor (Oxford Optronix, Abingdon, United Kingdom). The probe was pre-coated with a 10 U/ml solution of heparin in saline to prevent hematoma, then slowly inserted into the vastus intermedius muscle along the track of a puncture made using a 21G × 5/8″ Microlance needle to a depth of 5 mm. tPO2 measurements were continuously recorded using PowerLab 4/25 coupled to Chart 7 (AD Instruments, Oxon, UK).

At the conclusion of the protocol, animals were euthanized by severing of the aorta and subsequent exsanguination. Myocardial infarct size (IS) was subsequently measured as explained below.

### Determination of Infarct Size

At the conclusion of the protocol, the heart was either removed from the Langendorff apparatus (ex vivo experiments) or else isolated from the animal and the aortic root cannulated (in vivo experiments), and 5 mL of 1% 2,3,5-triphenyltetrazolium chloride (TTC) in phosphate-buffered saline injected through the aortic cannula and incubated for 10 min at 37 °C in order to demarcate the infarcted (white) versus viable (red) tissue [11]. For in vivo experiments, the LAD coronary artery was re-ligated in order to perfuse Evans blue dye (2 mL of 0.5%) to delineate the area at risk (AAR). After the incubation, hearts were frozen overnight at − 20 °C and sectioned perpendicular to the long axis the day after, being the slices transferred into 10% neutral formalin buffer for 1 h at room temperature. Images were taken and evaluated through planimetry analysis using Image J version 1.47 (NIH, Bethesda, MD) to quantify myocardial IS as a percentage of the AAR.

### Statistical Analysis

All data were pooled in the same unit of analysis (percentage of IS over AAR) and shown as mean (SEM). For each independent experiment, we calculated the effect size as a raw difference in means (the means of the experimental group, either IPC or RIC, minus the mean of the control group) of the percentage of myocardial IS and corresponding 95% CI and used linear regression models to establish comparisons between groups (IPC vs. control for five independent experiments and RIPC vs. control for three independent experiments). To estimate the overall effect of each intervention with higher accuracy, we used random effect models, in which some heterogeneity is allowed, and take into account the precision of individual experiments and the variation between experiments (between-experiment variation), weighting each experiment accordingly to obtain an overall estimate.

The robustness of our findings was tested in a sensitivity analysis by performing an additional analysis using the standardized difference in means (SMD; the mean of the control group minus the mean of the RIPC group, divided by the pooled SD of the two groups) [4].

The two-tailed significance level was set at *P* < 0.05. STATA software version 13.1 (Stata Corp, College Station, TX, USA) and GraphPad Prism version 6.00 (GraphPad Software, La Jolla California, USA) were used to perform the analyses and produce the graphs.

## Results

### Local Ischaemic Preconditioning

**Fig. 2:**
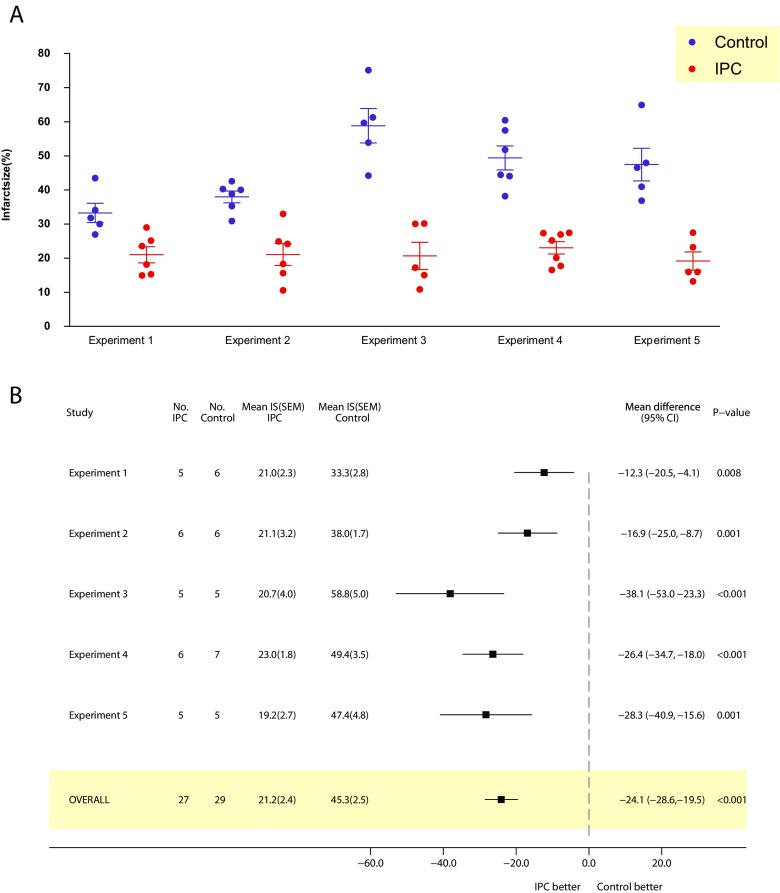
Effect of local IPC on myocardial infarct size in the ex vivo mouse model of IRI. Panel **a** displays raw data for each independent experiment. Panel **b** depicts a forest plot where left side of the *x* axis favours IPC and the right side favours control. An overall beneficial effect of IPC was observed. Crude mean difference are reported for each independent experiment, whilst the combined mean difference estimated through a random-effects model is reported for the overall estimate. The random-effect model provides an accurate estimation by weighting each experiment according to their precision and allowing for some expected random heterogeneity (between-experiment variation) inherent to all biological processes

The analysis contained five independent experiments, including data for 27 control mice and 29 animals that underwent IPC. Overall, IPC reduced infarct size in the area at risk by a mean difference of 24.1% (95% CI 19.5 to 28.6%) when compared to untreated controls (*P* < 0.001) (Fig. 2). Notably, IPC treatment effect was consistent across all experiment sets, and some between-experiment variation was observed (the minim effect size was 12.3%, whilst the maximum effect size was 38.1%). Importantly, between-experiment variance was taken into account to estimate the overall treatment effect.

### Remote Ischaemic Preconditioning

**Fig. 3:**
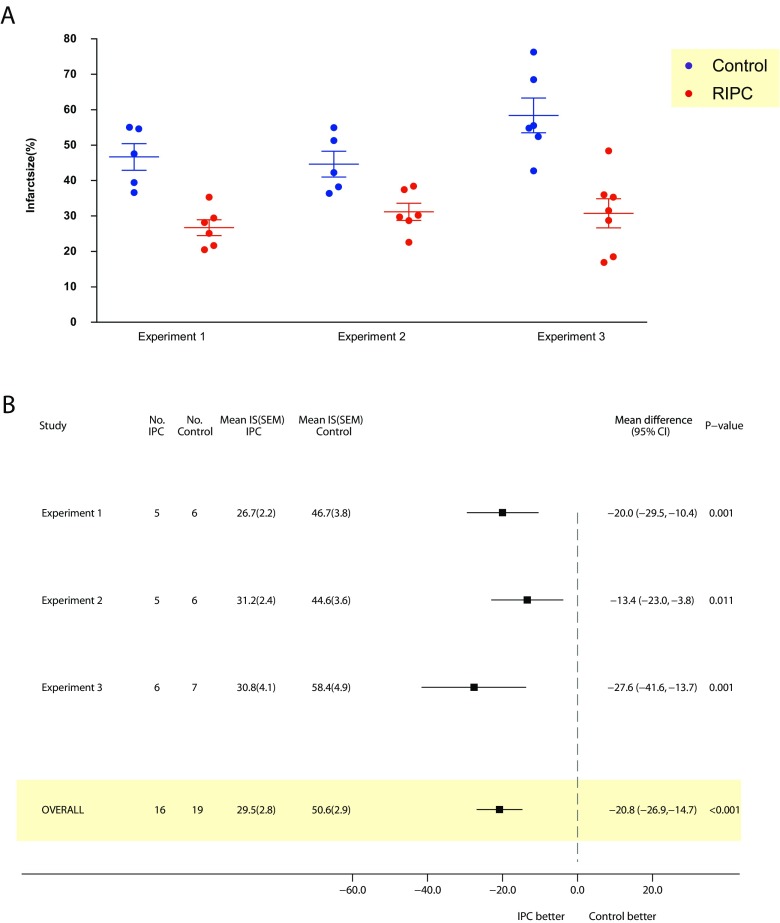
Effect of RIPC on myocardial infarct size in the in vivo mouse model of IRI. Panel **a** displays raw data for each independent experiment. Panel **b** depicts a forest plot where left side of the *x* axis favours IPC and the right side favours control. RIPC showed an overall beneficial effect. Crude mean difference are reported for each independent experiment, whilst the combined mean difference estimated through a random-effects model is reported for the overall estimate

The analysis contained three independent experiments, including data for 16 control mice and 19 animals that underwent RIPC. Overall, RIPC diminished infarct size in the area at risk by a mean difference of 20.8% (95% CI 14.7 to 26.9%) when compared to untreated controls (*P* < 0.001). Notably, RIPC treatment effect was consistent across all experiment sets, although some heterogeneity (between-experiment variation) was observed: the treatment effect ranged between 13.4 and 27.6% (Fig. 3).

### Sensitivity Analysis

When re-running our analysis using the standardized mean difference (SMD), all results were similar to those found using the weighted absolute difference in means. We found a highly significant (*P* < 0.001) overall effect of IPC (SMD of 6.3; 95% CI 4.07–8.43). Similarly, we found a highly significant (*P* < 0.001) summary SMD of 5.49 (95% CI 3.91, 7.08) for RIPC. Of note, the magnitude and direction of treatment effect for both IPC and RIPC, as well as the level of significance did not change substantially.

## Discussion

Our present data indicate that local IPC reduces myocardial infarct size by a mean difference of 24.1% (95% CI 19.5 to 28.6%) and RIPC diminishes myocardial damage by a mean difference of 20.8% (95% CI 14.7 to 26.9%). Notably, the direction of the results was consistent across sets of experiments, and the overall estimate remained consistent in a sensitivity analysis using standardized mean differences. In this study, we pooled the results from eight separate experiments testing for two interventions carried out in our Institute over the last 3 years in order to obtain precise estimates of efficacy.

Efforts to avoid bias and random error are as important when reviewing the results of experimental models as when reviewing the results of human studies, given that this evidence would be used to determine which interventions are taken forward in clinical trials. This would apply for all cardioprotective interventions which can potentially be translated to the clinical setting and would imply that further efforts are needed to accurately estimate treatment effects discounting both the effect of chance and biological variability, before concluding that these results can be translated to the clinical setting thought a randomized trial. Notably, previous meta-analysis in different fields of preclinical research shows that publication bias and methodological flaws are often responsible for systematic effect size overestimation and thereby incorrect conclusion about efficacy [1]. Thus, it is needed to estimate treatment effect using standardized protocols in relatively homogenous experimental conditions. Using data already collected is in accordance with the principles of the 3Rs (reduction and replacement of animals and refinement of procedures), which represent the cornerstone of sensible and streamlined animal research [12].

Since the clinical feasibility of local and remote ischaemic preconditioning in IRI is mainly limited to elective intervention, such as interventional or surgical coronary revascularization, we believe that our results have implication in other equally important aspects. We expect these findings to be used as the standard for other cardioprotective interventions to determine by how much myocardial IS can be reduced following an IRI insult. Our observations may contribute to more successful development of new therapies aimed to reduce myocardial IRI, as cardioprotective therapies providing a limited treatment effect in comparison with expected IS reduction provided by the gold-standard (IPC or RIPC) should be re-assessed before moving the research towards the clinical setting. Moreover, taking into account that our estimate effect size is the result of pooling more than 90 animals in a single dataset, we believe that these results are of potential use when establishing positive controls within a set of experiments. IPC has become the paradigm to study molecular signalling in cardioprotection [8, 10, 13] and researchers can use our values to compare whether their IPC models sufficiently reduce myocardial infarct size or whether they need to change the protocol to obtain a more suitable model. Small rodent animals of AMI have been extensively used over the last decades to elucidate the pathologic responses to IRI, and their use has provided substantial insights of the molecular signalling underlying most of the cardioprotective interventions, thus unveiling potential drug targets.

The magnitude of infarct size reduction using either IPC or RIPC estimated at individual animal-level in our study is similar to those reported in previous meta-analyses using aggregated data in highly heterogeneous backgrounds [4, 6, 14]. Most of these studies lack the consistency of using a single standardized protocol, as most of them have pooled data from both local and remote conditioning and have pooled together results from different protocols (i.e. number and duration of cycles), and some of these results have been reported for different organs (i.e. heart, kidney). Notably, our results define with precision the magnitude of the effect of the protection afforded by IPC in an adequately-sized subset of experiments in two well-defined and recognized models using standard protocols consistently in the same organ.

Given that all the experiments were performed in the same laboratory, we observed less heterogeneity in our statistical analysis in comparison with previous meta-analysis of pre-clinical studies. This is a double-edged sword, as in one hand we obtained more precise estimates, but on the other hand, this should be understood as a limitation to generalize our results. Moreover, caution should be taken when extrapolating our findings to other settings, given that we used young animals with no comorbidities. The failure to translate cardioprotective therapies into the clinical setting has been attributed to a disconnection between animal models and the clinical setting [15]. It is important to appreciate that our study was not designed to address this disconnection. However, it would be important to undertake similar studies to that of the above, using comorbid animals to better understand their influence on myocardial infarction.

In conclusion, using a relatively large animal data set, we found that local IPC reduces myocardial infarct size by 24.1% and RIPC by 20.8% in the ex vivo and in vivo mouse models of IRI, respectively. These values may become reference values for other researchers to either establish their positive controls or to determine by how much myocardial infarct size can be reduced by novel cardioprotective interventions following an IRI insult.
